# Awareness and Awakening: A Narrative-Oriented Inquiry of Undergraduate Students' Development of Mindful Agency in China

**DOI:** 10.3389/fpsyg.2017.02036

**Published:** 2017-11-21

**Authors:** Qing Wang, Ho Chung Law, Yan Li, Zhanfei Xu, Weiguo Pang

**Affiliations:** ^1^School of Psychology and Cognitive Science, East China Normal University, Shanghai, China; ^2^Cambridge Coaching Psychology Group, Cambridge, United Kingdom; ^3^Colombo Institute of Research and Psychology, Colombo, Sri Lanka

**Keywords:** mindful agency, coaching psychology, narrative analysis, undergraduate students, intervention

## Abstract

The article explores undergraduate students' experiences of developing mindful agency as a positive learning disposition, their perceived change as a learner, and the possible impact of mindful agency coaching on students' learning and personal growth, using a narrative research method. Seventy Chinese undergraduate students generated personal reflective journals and eight participants' journals were selected to enter into the narrative-oriented inquiry. Our analysis revealed a number of primary themes based on which we produced a meta-story. The supplements of the story were exacted for further critical cross-case discussion. The finding indicated that the multifaceted development of mindful agency involved learning methods, emotional regulation, strategic thinking, and awareness of planning, openness to experience, self-acceptance and self-esteem, and learning engagement, with enhanced sense of personal awareness and awakening. The coaching was supportive for students to foster positive self-identities and become more reflective, mindful, and self-determined.

## Introduction

This narrative study forms part of a larger project which aims to design, implement, and evaluate an innovative intervention program to develop undergraduate students' positive learning dispositions in China. What learning dispositions count as essential for students and how educators can enhance them have inspired an extensive range of studies in educational psychology including motivation, learning strategies, attitudes (Ralph and Markus, 1995; Riding and Rayner, 1998; Crichton and Kinsel, 2003; Howell and Watson, 2007; Cassidy, 2011; Dermitzaki et al., 2013; Velayutham and Aldridge, 2013). In this study, we focus on an emerging concept of learning disposition, mindful agency, and aim to gain in-depth knowledge of how it develops. More specifically, we developed a coaching program based on narrative, positive psychology, and mindfulness coaching approaches. We invited the students to keep narrative journals throughout the coaching program. After the completion of the program, we collected their narrative journals and conducted narrative analysis in order to understand participants' experiences of coaching.

Our research questions are:

1. What are the experiences of the student participants in terms of learning and personal development?
2. How do the students perceive themselves as learners throughout the coaching program?
3. What is the impact of the mindful agency coaching on students' development?

In doing so, we could understand better the development of mindful agency and how students perceive their growth from their experiences, using a narrative research method. The concept of awareness and awakening emerged from our analysis and are expanded further in the discussion.

### Mindful agency and learning

Whilst mindfulness has become a popular practice in the West (Brown and Ryan, 2003; Kabat-Zinn, 2003, 2005), mindful agency is a relatively new concept. Mindful agency is defined as “*a positive learning disposition that involves a strong sense of autonomy, authenticity, competence, intentionality and self-awareness in observation, management and regulation of the motivational, cognitive, emotional, and social processes that an individual needs to be able to engage in effective learning in order to achieve his or her own learning goals”* (Wang and Peng, 2017). The original concept of mindful agency was coined by Deakin-Crick et al. (2015) in their reanalysis of 15 years' data derived from the project of learning power using Effective Lifelong Learning Inventory (Deakin-Crick et al., 2002a,b, 2004; Deakin-Crick and Yu, 2008). The construct of mindful agency is associated with three factors: (1) managing the process of learning, (2) managing the feeling associated with challenge, and (3) agency in taking responsibility for learning purposes, processes, and procedures. Mindful agency demonstrates significant positive correlation with creativity, curiosity, sense-making, optimism, and hope measured in the learning power model (Deakin-Crick et al., 2015). Early scholars suggested that mindful agency conceptually integrates various learning dispositions and capacities, such as meta-cognitive strategies (Veenman et al., 2006; Vrugt and Oort, 2008), emotional intelligence (Varela et al., 1991; Fredrickson, 2001; Fredrickson and Losada, 2005), self-efficacy (Bandura, 1982, 1986, 1994; Zimmerman et al., 1992; Zimmerman and Schunk, 2006), and hope theories (Snyder et al., 2002).

Wang and Peng (2017) extended the notion of mindful agency by explicitly connecting agency theories (Carter and Sealey, 2000; Hazel et al., 2008; Bown, 2009; Gao, 2010; Adele et al., 2011), hope theories (Snyder et al., 2002), self-determination theory (Deci and Ryan, 1985, 2000; Koestner and Losier, 2002), flow (Csikszentmihalyi, 1990; Engeser et al., 2005), mindfulness (Langer, 1993, 1997, 2000; Brown and Ryan, 2003; Kabat-Zinn, 2003, 2005), and acceptance and commitment therapy (Hayes, 2004; Hayes et al., 2006). Further, they revised the comprehensive psychometric tool and identified a five-dimension structure of mindful agency by exploratory and confirmatory factor analysis using two different cohorts of Chinese undergraduate students. The five factors of mindful agency involve *learning methods* (the ability to adopt different methods accordingly and flexibly in various learning activities), *emotional regulation* (the ability to regulate negative emotions when facing obstacles and challenges), *awareness of planning* (the ability to be aware of and adopt appropriate strategies when approaching particular tasks), *openness to experience* (being open and acceptive to various learning experiences, acknowledging them as natural parts of the learning process), and *learning engagement* (deep immersion, active participation, and profound intrinsic motivation in learning). Empirically, significant results have been found that mindful agency is positively correlated with creativity, particularly with fluency and originality (Wang and Hu, 2016), positively correlated with academic emotional regulation ability (Wang and Zhang, 2016), and learning strategy regulation ability, mediated by self-efficacy (Wang and Ar, 2016), and negatively correlated with academic procrastination, mediated by self-regulatory ability (Wang and Yu, 2016).

### Mindful agency coaching

Mindful agency is an emerging and important concept of positive learning disposition. We have been developing various coaching interventions to help students to enhance their mindful agency over a number of cohorts. The framework of the coaching program reported in this study integrated narrative, mindfulness, and positive psychology to develop mindful agency in the undergraduate students. The narrative coaching addressed mindful agency's central component, i.e., the sense of ownership and responsibility in one's learning. Narratives enable individuals to forge new connections between their stories, identities, and values in order to generate action and embody new options (Drake, 2007, 2010; Law, 2007; Stelter and Law, 2010; Kerr et al., 2013; Stelter, 2013). In mindfulness-based coaching, we adapted Mindfulness-Based Stress Reduction (MBSR, Kabat-Zinn, 1990) and Mindfulness-Based Cognitive Therapy (MBCT, Segal et al., 2012) into a series of activities that were suitable for undergraduate students. It would exert impact on how individuals perceive their learning experiences from different perspectives, regulate and manage emotions when they face challenges, and flexibly respond to different learning occasions. The positive psychology coaching (Seligman et al., 2006; Biswas-Diener, 2012), as a strength-based intervention, would help students to explore their core values, resources and motivations when they are engaged in learning. In summary, the mindful agency coaching program was designed to support participants' self-directedness and intrinsic motivation in learning, help them to develop a sustained, non-discriminatory observation of momentary experience, promote the awareness of their own thoughts and emotions, reduce their emotional and behavioral reactivity, and encourage a non-judgmental and non-attaching attitude (Passmore and Marianetti, 2007; Spence, 2008; Cavanagh and Spence, 2013). Thus, the students would develop mindful agency. The quantitative measurements of the effectiveness of the coaching program are reported elsewhere.

### Narrative analysis of student learning

This study aims to understand the development of mindful agency and how students perceive their growth from their experiences during an innovative intervention program. Compared to other qualitative research methods, such as grounded theory (Glaser and Strauss, 1967; Fassinger, 2005) or interpretative phenomenological analysis (Smith and Osborn, 2015), narrative analysis enables us to better understand how individuals make sense of themselves (self-identity) and of their lived experiences through their stories (Polkinghorne, 1995). Narrative analysis approaches have been extensively used in educational and psychological studies (e.g., McAdams et al., 2006; Hiles and Cermak, 2008; Mangione et al., 2013; Moss and Pittaway, 2013; Murray, 2015). These studies had in common with our study that we explored a holistic picture of how different experiences, realities and self-identities are constructed, deconstructed and re-constructed, and discovered and explored students' learning identities more effectively. The novelty in our study was that we used students' personal reflective writing as evidence to see if there existed any change during or after the coaching program.

There are a wide range of narrative analysis approaches. We adopted the narrative-oriented inquiry (NOI) (Hiles and Cermak, 2008; Hiles et al., 2009) as our research method. It provides us with a more formal structure and detailed procedure for the analysis, and captures the meaning in relation to the key issues to understand participants' identity positions (IP), as exemplified by the work of Law and Basil (2016). Six interpretive perspectives are used in NOI: *sjuzet—fabula* (essential), *holistic content, holistic form, categorical content, categorical form*, and *critical narrative analysis*. The approach makes distinction between *fabula* (a sequence of events that occurred, i.e., what are told in the stories) and *sjuzet* (i.e., the way in which the stories are told by different narrators). By analyzing the *sjuzet* carefully (e.g., terms, tones, words, phrases, and other forms of expression that students used to express or indicate their individual thoughts and emotions), we could see how the participants construct the meaning of events happening in their lives, thus revealing their values and identity positions (see Law and Basil, 2016). We used students' personal reflective writing as narrative data to obtain an in-depth knowledge of participants' learning attitudes and sense-making processes beneath learning-related activities. Besides, reflective writing is regarded as an effective transformative pedagogical strategy that can be applied in variety of educational settings to support students' sense-making of their experiences and improve self-awareness and learning agency (Moon, 1999; Babcock, 2007; Hume, 2009; Barney and Mackinglay, 2010; Waston, 2010; Knight, 2011; Hyland-russell, 2014). Moreover, narrative tone is a key concept in the narrative analysis which is concerned with the overall emotional flavor of the narrative (Murray, 2015) with three primary structures of narratives (i.e., progressive, regressive and stable) (Gergen and Gergen, 1986; McAdams, 1993; Crossley, 2000). We identified the narrative tone by analyzing the emotional thread of the whole segments and any emotional changes as the story unfolded.

## The data in context

### Participants

The participants were recruited by responding to an advertisement on the mindful agency coaching via posters, emails, websites, text messages, and snowballing among undergraduate students in a university located in East China. There were 99 undergraduate students who initially signed up. There were 70 students (Female = 60, Male = 10, *Mean Age* = 20, *SD* = 2.4) who completed the whole coaching program, and 29 students who withdrew at different stages in the coaching.

### The mindful agency coaching process

The participants were asked to join in an 8-week group-based mindful agency coaching program (see Table 1 for details). The program included 4 bi-weekly sessions of 3 h each. The coaching was designed and delivered primarily by the first author, a Chartered Psychologist and Licensed Coaching Psychologist, with the help of four research assistants. All the participants were provided a notebook that was intentionally divided into two columns. The left column was for note-taking of spontaneous ideas, opinions, feelings, and answers to questions during the workshops. The right column was for reflective notes on their most important learning activities and the associated emotions between workshops, offering additional self-discovery. The journals could be created in any form—some participants wrote texts in both Chinese and English, and some integrated texts and pictures.

**Table 1:** Mindful agency coaching program.

**Session**	**Focus**	**Main activities**	**Aims**
Session 1 3 h	Mindful agency and learning	Introducing the concept of mindful agency Explicating the relation between mindful agency and learning Exploring students' values, reasons, motivation, and resources in learning	Foster a sense of agency, ownership, and responsibility Enhance intrinsic motivation
Session 2 3 h	Mindfulness-based coaching	Explaining mindfulness and its characteristics Preparing the attitudes of mindfulness practice Bodily sensations; mindful breathing; mindful moving; body scans; eating, sitting, and walking meditation; loving-kindness meditation Making personally tailored mindfulness practice	Develop self-awareness Deepen reflexivity Cultivate self-management and self-discipline
Session 3 3 h	Narrative-based coaching	Discussing storytelling and narrative psychology Personal narrative: writing a letter for yourself in 10 years Narrative in pairs: reconstruction of stories and self Group narrative: A journey to the West	Develop learning engagement Cultivate a sense of goal, aspiration, and hope Develop self-directed and collaborative learning ability
Session 4 3 h	Reviewing, sharing, and reflecting on the coaching	Exploring generative moments in learning Revealing character strengths Sharing experiences of MA coaching Self-evaluation of growth as a learner	Foster self-knowledge and self-awareness Explore personal resources Track the progress of learner's identity

### Collection of narrative data

Seventy participants' personal reflective journals were collected immediately after the coaching program. The journals were returned to the participants who would like to claim their journals after the completion of data analysis.

### Ethical considerations

This study was carried out in accordance with the recommendations of Ethics Guidelines of Research Ethics Committee at East China Normal University with written informed consent from all participants. All participants voluntarily joined in the mindful agency coaching and gave their written informed consent in accordance with the Declaration of Helsinki (Inc, 2009). The research protocol was approved by the Research Ethics Committee at East China Normal University. English pseudonyms were used in order to protect participants' identities. All the narrative data was kept confidentially and only used for research purposes. Given the nature of narrative analysis (co-authorship), researchers, and participants shared the ownership of the stories that generated from the analysis.

## Analytical procedure of NOI

For an in-depth NOI analysis, we purposely selected students' personal reflective journals that met the following criteria: (1) they were considered of satisfactory completeness and continuousness (e.g., contained personal notes of all the coaching sessions and regular diaries during the intervention); (2) they were relatively coherent and clear with identifiable storylines, events, and characters; (3) they were of great richness and reflexivity (e.g., contained detailed reflections from personal and multiple perspectives); and (4) they were considered highly creative, using consensus assessment technique (CAT) on creativity (Amabile, 1982, 1996; Amabile et al., 1994) among five researchers. We excluded journals that (1) only copied down the content of Power Point documents in the coaching program; (2) did not contain reflective diaries; or (3) contained blank or missing pages that showed an incontinuous pattern of diaries. There were eight reflective journals that met the inclusion criteria and entered into the analytical stage using the following analytical procedure:

1. Conduct an overall reading of all the data many times until one gets a sense of the storyline, narrative structure, and the general themes.
2. Write a brief summary or abstract to describe each participant's story.
3. Conduct a line-by-line analysis to identify a sequence of interrelated *fabula* and highlight the *sjuzet*.
4. Identify the linkages and relationships between different themes, plots, and sub-plots.
5. Analyze the story using a coding frame (adopted from Labov and Waletzky, 1997) in accordance to the good practice suggested by a range of literature in narrative methods (Hiles and Cermak, 2008; Patterson, 2008; Hiles et al., 2009; Bold, 2012).
  - Abstract (Abs)
  - Setting
  - Complication (Comp, complicating action referring to a concrete scenario, e.g., then what happened)
  - Evaluation (Eva)
  - Result (Res)
  - Coda (referring to a conclusion or ending)

The narrative analysis was carried out using the following coding notation:

*Sjuzet* is underlined e.g., Feeling life is hatred
Fab 1—Start of *Fabula* 1, etc.
IP 1—Identity Position 1, etc.
[.!]—Omissions, identifiers removed, comments, etc.

The above coding frame enabled us to identify how the participants described various significant moments (e.g., crises, tuning points, redefining goals), which were linked to their values, beliefs, and identity positions.

6. Analyze different individuals' narrative texts to discover their own stories, and compared the identified categories, themes, and plots across each story.
7. Construct a typical story (meta-story) which embodied holistic, significant patterns that captures the meaning of most stories. Any exceptional stories that were not captured by the meta-story are included in the discussion where appropriate.
8. Identify segments in individual's stories that were different from or supplementary to the meta-story, and analyzed how these segments contributed to our understanding of particular experiences.

We arranged the transcripts on the left-hand side and made notes and comments in the right-hand column of each page when we coded the data. The same narrative analysis method was conducted by different research assistants respectively, and then compared, discussed, and revised together to obtain consensus and inter-subjectivity. We provided an example of data analysis in Table 2.

**Table 2:** Narrative analysis of Susie's story.

Abstract: Susie, a 21-year-old undergraduate student who viewed herself procrastinating, tried to be more structured and focused in learning and become an efficient and creative learner. She thought that mindful agency coaching program was an opportunity for her to develop a more positive learner's identity.	
Monday 11 April Segment 1: Most time I was out of order. I attended class in the morning from 10:00 to 11:30. At other times I was sleeping, feeling I had nothing left to live for, no energy, and no motivation. I saw myself useless, unwanted, and had no effort to take care of others' feelings.	Fab1 Setting1 Comp1 Eva1 Res1 Coda1 → IP1 [evaluate general negative feeling about life to one own negative self-identity. Low self-esteem. Pessimistic, regressive tone]
Segment 2: In the evening, I cheered myself up to join the choir. [I was] focused and singing well when feeling good about myself (though only lasting for a short while).	Fab2 Setting2 Comp2 Eva3 Res2 Coda2 → IP2 [a sense of struggle summing up the energy with a more positive self-belief.] Plot argument
Segment 3: [I was] learning to sing, worrying that I would fail to sing well and make the right intonation. [I was] very confused, and cannot make the right sound when nobody sang around me. I gradually got some feel for it and tried to match my voice to the chord, but still felt fickle and diffident. [I should] try to use a learning strategy to record my own voice and practice more after class. [I believe that] “Diligence can make up for lack of intelligence”.	Fab3 Plot argument Eva3 Res3 Coda3 IP3 demonstrates learning, solution focused, ends with a [optimistic, progressive tone]

The analysis was conducted in Chinese. After the analysis, the preliminary findings were sent to the participants for review to ensure the accuracy. The results were returned with minimum corrections and comments. For inter-subjectivity, the results were further cross-checked and peer-reviewed, and translated into English for the purpose of publication. We consulted English native and bilingual peers to check accuracy and clarity, making sure that the English texts fully conveyed participants' original ideas and our analysis without distortion.

## Results

### *Sjuzet* and *Fabula*

Owing to the limited length of this article, here we provide an example of the analysis from one participant (called Susie) to show how *fabula* (i.e., a sequence of events that occurred) and *sjuzet* (i.e., the way in which events are told) served as integrative accounts of her everyday life and development of mindful agency (see Table 2). As presented in the table, *fabula* consisted of Susie's school activities, including attending classes, joining in the choir, and practicing singing. *Sjuzet* did not emerge from the narrative texts directly, instead, it consisted of the words and phrases that revealed feelings, deep thoughts, and self-identity positions, based on the coding frame in NOI.

### Holistic-content

The perspective of holistic-content established the links across all participants' shared stories and combined them as a whole. Four researchers in our team read through all the journals, and selected 13 episodes that covered participants' common experiences and associated feelings in their narratives during the workshop and daily activities sequentially. Then we organized these episodes and weaved them into an original meta-story, which consisted of character, plot, prelude, and coda in a typical story. The pseudoname Linda was used as the character.

*My name is Linda. I am an ordinary 2nd Year college student. My life seems to be filled with different kinds of learning and social activities. However, I really don't know what exactly I am busy about. I am a bit passive in learning, and my learning efficiency is rather low. Procrastination is my problem. I have been trying to get rid of this bad habit many times but failed. Apart from being passive, sometimes I am also impatient when interacting with others. I'm not satisfied with my life. I really hope that I could be someone who is highly efficient, purposeful and motivated, and able to concentrate on learning, never procrastinate, and know how to easily get on well with others*.

*One day I noticed a poster about mindful agency coaching in the university and thought: I could make some changes through this opportunity and decided to have a go. This was the first time that I learned the concepts of mindful agency and mindfulness, and their significance to my learning. I found myself actually have a lot of strengths that I hadn't noticed before, such as curiosity. The coach asked us to record the best and the worst learning conditions that happened to us every day, which was very new for me. I just found it interesting and novel to write down my thoughts and feelings about my experience in such a coherent and clarified way*.

*The 2nd session was very interesting. It provided us with various methods to apply the mindfulness-based practice. I realized that I was able to get in touch with my own body, thoughts, and feelings. After this session, I often exercise mindful practice in my daily life. I practice mindful breathing when I am waiting for my friends, or when I feel stuck in writing assignments. Then I can calm down and just allow ideas flow in my mind that help me. Sometimes I have the “A-ha” moment. By reviewing my journal, I am pleased to find out small progress each day: I play with my mobile phone less often when studying (I am very proud of that!) and become more focused on one thing at a time rather than doing many things simultaneously. I am more aware of my emotional state in each present moment and become more rational and composed when I face difficulties*.

*In the 3rd session, I wrote a letter to my future self (in 10 years later). I tried to imagine what my life, career and family would be like and felt so close to myself. I felt a strong sense of hope and became more aware of my life goals. In the collaborative narrative, I found that I could re-organize my thoughts via communication with others. Meanwhile, I felt being trusted and supported. I was able to put myself into others' shoes during the process of communication and understand others' issues more empathetically*.

*When I looked back at myself in the summary session, I realized that I have changed gradually, quietly, yet significantly. I learn to accept “me-at-the-present” and do not burden myself with huge goals. I accept myself as a unique individual and I don't need to be perfect in others' eyes. I learn to be at peace with my own feelings and thoughts, to live with these ideas, no matter they are positive or negative. I extend this acceptance to the interaction with peers… I take initiatives in learning by discovering what I am really passionate about. There are always ideas and thoughts emerging in my mind. Although sometimes I am distracted by them, I am able to allow these thoughts gently come and go, like butterflies, without judgment. I am able to concentrate on tasks using more appropriate learning strategies. I am able to notice and comprehend my own conditions during learning, knowing when to push myself a bit harder and when to relax… I am a learner, not just a student, and this growth journey continues*.

As the meta-story demonstrates, participants reported that they perceived mindful agency coaching as a self-enhancing experience. These are common changes during the process. The four workshops linked their experiences together and improved their mindful agency in terms of increasing self-acceptance, self-esteem, self-awareness, and reflexivity, adopting multiple perspectives of viewing oneself in relation to others and things in their environment, paying more attention to the positive aspects and managing distractions. They became more focused and mindful in their own learning and more patient and loving toward others.

### Holistic-form

The perspective of holistic-form focused on the structure of the overall narrative, the plot that was threading through *fabula*. Firstly, the *fabula* was summarized in short phrases. Second, we clarified the settings of certain events occurred. Third, we organized the *fabula* chronically according to participants' journals. Fourth, we established links between different events in different settings. We provide an example in Table 3. Cathy's Fab1 reflected everyday learning activities without mindfulness to Fab4, her low efficiency during study such as distraction in Physics class, procrastination when doing homework and so on. This story was interpreted by her failure to regulate negative emotions and strong competitive tendency when comparing herself with others, and so to the point of making herself feel irritated and regretted when reviewing before the final exam. In viewing the holistic structure of narratives, we could see how particular events and experiences were connected and what kind of relation that these events represented.

**Table 3:** Cathy's story.

Fab1/Setting1, everyday learning activities without mindful agency [•Res1 pessimistic (regressive) tone] Fab2 doing homework Fab3 cannot focus on reviewing Fab4 attending Physics class Fab5/Setting2, applying mindful methods to learning [•Res2 optimistic progressive tone] Fab6 choice time Fab7 reviewing physics and doing experiments with curiosity—link to (Fab3) Fab8 making study plans—link to (Fab4)
……
Fab51/Setting17, generate moments Fab52 situation and function Fab53 chatting or regulating emotions to rebuild it Coda optimistic progressive tone

### Critical analysis of categorical form and content

This section shows the critical analysis of each participant's narrative and presents the segments that are different from or supplementary to the meta-story. According to Hiles (2007), our narrative identities are grounded on a series of inter-related identity positions. We can identify the multiple identity positions of each narrator from the unfolding reflective stories (Law and Basil, 2016). Although the participants shared common experiences in their stories, they positioned themselves with respect to their own sense of self in the context of change and growth as learners and overcame a series of challenges that came along with the change.

The extent of the mindful agency development varied in different individual participants. Taking learning engagement as an example, Yale could concentrate on his current task for a long time after the coaching, while Ivey succeeded in excluding the distraction but still multi-tasked sometimes. Although participants' previous learning status varied before the coaching, they seemed to achieve an inner balance by using distinctive learning strategies throughout the mindful agency coaching program. For instance, Zoe used to be easily distracted in learning, and the mindfulness practice helped her to become more focused and improve her learning efficiency; while Cathy used to be overburdened, and she became a learner who knew how to combine exertion and rest with mindfulness-based methods. Winnie was a student enrolled in liberal art and she implemented mindfulness approach into reading and writing and achieved a state of flow. Ivey used positive reinforcement to link positive stimuli and learning activities in order to become more motivated to learn Japanese. Moreover, Winnie and Ivey enhanced their awareness of planning with to-do lists each day, alleviated procrastination and increased their learning efficiency; they felt a sense of contentment and autonomy.

Some segments from individual participants were different from the meta-story. For example, Yale and Donner still viewed themselves as perfectionists after the mindful agency coaching; however, their tone became more optimistic and they were willing to accept this characteristic, as Donner wrote:

*There is always a drive inside me to pursue perfectionism, even though I can accept myself being imperfect*.

According to Ma and Zi (2015), there are two types of perfectionism: negative perfectionists are closely related to fear of failure, high objective and strict norm; positive perfectionism has strong relationship with positive self-evaluation and self-expectation. The perfectionist disposition can be beneficial if students learn to embrace this characteristic and transform it into a positive type.

## Discussion

This paper has offered an innovative perspective to investigate Chinese undergraduate students' experiences of developing mindful agency by participating in a coaching program, particularly designed in mindfulness-based, narrative, and positive psychology coaching approaches.

Overall, we have found that most participants' stories were progressive with an optimistic tone. Participants portrayed the experience of developing mindful agency as an opportunity for self-exploration, advancement, and growth. They all started with different personal learning issues. Throughout coaching, they took new perspectives in understanding their values, motives, and resources in learning, developed strategies of integrating what they had learned in the coaching into their daily life, increased self-awareness, and fostered a healthy learner identity.

The most distinctive observation in the study is that through the narrative journey, participants' identity positions have shifted. At the beginning, the students were generally very stressful and critical about themselves; they have tried to study hard, trying to please their teachers or win respect from their peers without success. Through the coaching process, they gradually became more autonomous and self-determined, initiating learning behaviors from a sense of personal meaning and volition. This may be referred as motivation regulation when the value of an instrumental behavior has become congruent with one's sense of self (Deci and Ryan, 2000; Koestner and Losier, 2002). Also, it connected to hope theories (Snyder et al., 2002) because both a sense of agency (taking an active role in learning) and pathway thinking (searching methods to learn better) were observed. For instance:

*I used to studied hard in physics in order to get my teacher's recognition; to “save face”, I was afraid to ask my classmates to help. But now I can pay more attention to the process of learning itself and ignore the impact of the teacher's attitude toward my study. I care more about the pleasure of learning and learn to accept and reflect on the undesired results*. [Cathy]

In other words, the students became more open-minded and ready to accept who they were rather than trying to change themselves to fit into the ideal image of being a “good student.” This aligns with the core idea of acceptance and commitment therapy (ACT) (Hayes, 2004; Hayes et al., 2006). ACT advocates that one must mindfully accept and acknowledge what exists in reality for new perspectives and changes to occur. The students in our study learned to embrace themselves and became an authentic person, so that they developed self-appreciation and confidence, which empowered them to change and grow in their desired directions.

The overall growth of self-as-a-learner could be seen as parallel to the multifaceted development of participants' mindful agency in an upward spiral. The five dimensions of mindful agency emerged as the students developed and gained benefits from the coaching program.

### Learning methods and strategies

Participants integrated the methods and strategies learned in the coaching program, transferred them to their daily schedules and developed their own learning strategies, for example:

*I practice mindful meditation in the morning after I wake up in order to calm down my fretful heart, when writing papers and immersed myself in a state of flow… I make a to-do-list each day to increase my learning efficiency and live an organized life*. [Winnie]

By using these mindfulness-based strategies such as mindful breathing, students were able to immerse themselves in the present tasks and concentrate on their study for longer time. Sometimes they experienced the typical flow state (Csikszentmihalyi, 1990; Engeser et al., 2005) when they were totally absorbed into the tasks, forgot about the elapse of time, decreased a sense of self-consciousness, improved efficiency in performance and achieved desirable outcomes in their study. Some participants developed a sense of autonomy, self-discipline, and commitment by making to-do lists every day and following them through. This strategy helped students to alleviate procrastination and associated negative feelings such as shame or guilt, and fosters the ability of time management.

### Emotion regulation and management

Positive emotions, such as happiness, contentment, or joy, could strengthen students' self-efficacy, self-confidence, and trust in the strategies they used (Paul and Heather, 2000; Fredrickson and Joiner, 2002). Mindfulness was proposed to enhance awareness and broaden the scope of cognition to facilitate amplifying positive emotions (Tugade and Fredrickson, 2007; Hülsheger et al., 2013) as well as reappraisal of adversity (Garland et al., 2015). In mindful agency coaching, participants developed the ability of emotional regulation and management through two approaches: savoring positive experiences and accepting difficult experiences.

*I used to think others are so excellent and they had been devoted a lot to their study, and I was not as good as them…In fact, I don't need to be so despondent and deny myself. I learn to regulate my emotion by having a heart-to-heart talk with my roommates, or going go out to get some fresh air, appreciating the beauty of life. This helps me to become more confident, and I learn to discover and enjoy the pleasure of study*. [Cathy]

By perceiving, acknowledging and embracing negative emotions, the participants became more at ease with themselves. This attitude served as a protective factor for students from overly self-demand and self-critics when they did not fulfill expectations. By clearly differentiating “being mode” and “doing mode” (Kabat-Zinn, 1990), participants expanded their learning repertoire, became more reflective, and made conscious efforts to achieve higher learning goals.

I can sense myself in a bad mood and try to regulate negative feelings by doing mindfulness exercise… I tried not to react to the feelings immediately and took time to digest them. I try my best to do things, accept the results whether it is good or not, and reflect myself afterwards and expect to do better next time. [Cathy]

### Awareness of planning

The students became more aware of their goals, resources, and learning conditions. This could pave the way of becoming more able to make strategic plans of their study instead of being passively pushed by deadlines or other external forces. One participant wrote that she learned to select appropriate tasks according to her current condition:

*I tried to be aware of my condition and evaluate it: if I was energetic, then I would read the literature; if I was tired, I would listen to music to relax myself…I'm honest to myself and listen to my heart*. [Susie]

In addition, participants were better at time management and task organization with an increased awareness of planning. In the coaching program, participants were coached to observe themselves, monitor learning processes, adjust their own conditions and set proper goals for themselves. Writing reflective journals might also help the students in developing this awareness. Participants were asked to note down detailed experiences of critical moments in learning on a daily basis, e.g., when they were in their best mood, when they felt their energy was low, what their most important learning activity was, how they felt about it. The increased awareness of planning and self-regulated learning processes would lead to higher achievement (Eilam and Aharon, 2003).

### Openness to experience

Participants demonstrated a greater sense of acceptance and non-judgmental attitude to various learning experiences, more appreciation of others as who they are, and more patience in listening to different opinions. For example, Yale referred in his journal that he used to impose his own thoughts on others when he talked to people, and he hardly realized it. After he has developed mindful agency, he was more able to acknowledge different opinions and perspectives.

*I reflected on the reason why I felt unhappy: maybe I imposed my view on the others… I used to criticize a lot, and now I am able to keep my mind gentle and calm*. [Yale]

Research evidence has shown that mindful agency is positively related to curiosity and openness (Deakin-Crick et al., 2015), and mindfulness can reduce habitual responding to unconsciously acquired preferences by promoting a non-reactive and non-judgmental disposition (Whitmarsh et al., 2013). Participants in our study developed this sense of curious, non-judgmental attitude via guided instruction on mindfulness-based practice. Although formal practice of mindfulness usually takes 8 weeks (e.g., MBCT or MBSR) and our students only exercised for a short period of time each day (over the 8-week coaching program), they were encouraged to integrate mindfulness-based method into their everyday life and make personally tailored schedule. This could benefit a sustaining, long-term plan to develop mindfulness attitude and disposition.

Moreover, it seemed that participants have developed a balance between “being critical” and “being acceptive.” In higher education, one of the key educational goals is to develop students' critical thinking and reasoning, which often refers to the ability to challenge others' or one's own ideas and thoughts (James, 1987; Walker and Finney, 1999; Behar-Horenstein and Niu, 2011; Ghanizadeh, 2017). However, being able to trust and welcome authentic experience is missing in the larger educational picture. We addressed that an attitude of allowing and embracing experience is as important as healthy skepticism in thinking. Developing mindful agency might be a way to obtain that balance. Being self-aware can be extended to become aware of and respect others emotions, gaining social intelligence, and achieving social competence (Goleman, 1998; Law, 2013a,b).

### Concentration and engagement in learning

Participants enhanced their dispositions on concentration and engagement in learning throughout the coaching program. They were able to focus on their current learning tasks for a longer time, reduce mind wandering, and discipline themselves (refrained from playing mobiles, website browsing, using social media, or other distractive behaviors).

I try to use mindfulness methods to reflect my learning process by turning my mobile off and just concentrating on my current state… This process improves my learning efficiency and skills of time management, which helps me have a clear mind about my study. [Yale]

*Through the mindful agency coaching, I can chat with my friends attentively, consciously without playing my mobile phone. And at the same time, I pay more attention to the lecturer in the class without updating my posts in WeChat*. [Zoe]

There were two obvious benefits in this: first, it could help students to improve their learning efficiency and academic achievement by allowing more effective study time. Second, it could foster students' self-efficacy by successfully experiencing self-management and self-discipline. Mindful agency coaching might have a positive impact here that mindfulness could improve us in a deepened capacity for meaning-making and greater engagement with life (Garland et al., 2015) and positive psychology coaching could enhance engagement and flow in activities (Csikszentmihalyi, 1990; Seligman et al., 2006).

### Sense of awareness and awakening

In general, we found that participants perceived themselves as more autonomous, resilient, and self-determined learners throughout mindful agency coaching. The coaching program clearly has a positive impact on each dimension of mindful agency described above. Simply put, students have changed in this process. One participant, Zoe, wrote about the change in becoming a “happier learner”:

I was video-taped in other activities after the coaching. Someone said that he liked my smile, and today I see the whole process of my smiling. I change from being serious to happy. I see my smile more than twice a day and my mouth is curling up for a long time… I like my smile, and this makes me pay more attention to my own state and emotions. I like it when people around me can perceive these changes. [Zoe]

We wondered what exactly was underpinning the transformation. Perhaps these two phenomena for the awareness and awakening reflect the underlying changes. Awareness was greatly addressed in almost every activity in the coaching and reflective writing. When participants' awareness was broadened and expanded to themselves and others as well as to their learning environments, they could mindfully reappraise their current condition and improve it. We could see the evidence from students' stories that they obtained an enhanced awareness in self-observation from a reflective distance (critical, yet accepting), gained self-knowledge, understanding their values and motivation in learning, and self-enhancement, being willing, ready, and able to grow in their own learning journey. The increased awareness helped participants to identify their inner strengths and resources, which can be best described as having a sense of awakening. Awakening referred to the fact that participants were not “receiving” these attributes from the coaching program; they have already had those qualities within them, waiting to be unearthed via the process of coaching. In this sense, the coaching process can be described as “co-mining” with the coach and coachee. Mindful agency coaching supported the students to explore their hidden, potential, inner strengths, polish their existing skills, discover, and appreciate their internal and external resources, and become a kind of learner that they wanted to be. Thus, awareness and awakening are interdependent and interrelated, where an increased awareness would lead to an enhanced state of awakening and this awakening would further foster individuals' awareness, forming a virtuous circle (Pagnini and Langer, 2015; Donald et al., 2016).

## Concluding comments

The current study has adopted a narrative research method to explore undergraduate students' experiences of developing mindful agency as a learning disposition, their perceived growth and change as a learner, and the possible impact of mindful agency coaching on students' learning and personal growth. In our analysis, we combined both students' (“insider”) and researchers' (“outsider”) perspectives that offered multiple viewpoints with immediacy, intimacy, and yet holding a necessary critical distance for a scholarly investigation in order to balance detachment and involvement, thereby preventing researchers from “being lost” in one specific story being told by one particular narrator (Case et al., 2010). This may be at odds with the attitude of some narrative researchers (e.g., Reissman, 1993; Trahar, 2009). However, we remained mindful to maintain our critical stance as qualitative researchers and believe that this is an appropriate and balanced approach to offer the narrative account as evidence.

We are aware of some outstanding issues in conducting the research. First, reflective writing was complex and placed a high demand on students, therefore some participants without systematic coaching might find it difficult to integrate critical thoughts into a reflective journal (Ryan, 2011). The second issue is that some students were not comfortable with self-disclosure, particularly when there were different group members in each workshop (Wei et al., 2005). As a result, these participants might not benefit as much as others from the group narrative. Self-protective participants who are socially anxious tend to associate self-disclosures with decrease in liking and comfort (Meleshko and Alden, 1993; Wang et al., 2011). This may be overcome by a more gentle introduction to the coaching (e.g., an ice-breaking activity), and perhaps keeping the same members in each group. Third, the narrative analysis as a research method for impact evaluation was useful in capturing the richness of participants' stories and their lived experience and identity positions, but it did not provide quantitative evidence about the effectiveness of the program.

For the future research we recommend that quantitative measurements need to be developed for self-reflection to complement the qualitative approaches such as peer or coach's observations or interview. This would allow triangulation of the findings. When studying the implications of mindful agency coaching in different contexts and with different populations, we recommend a well-controlled intervention design and mixed methods could be used to validate the results and achieve triangulation in the conclusion.

For the future practice, three major implications could be drawn from our study in the context of higher education. First, mindful agency coaching could be used as a means of preparing students to deal with the challenges (such as anxiety and uncertainties) faced by them during their transition from secondary education to higher education (Kelly and Finlayson, 2010; Kyndt et al., 2015; Coertjens et al., 2017). Second, the self-scheduled mindfulness-based practices could be integrated into students' daily life with peer-guidance, coaching, mentoring, and monitoring. This would exert a sustained impact on students' psychological well-being and emotional balance (Passmore and Marianetti, 2007; Cavanagh and Spence, 2013). Third, mindful agency coaching program could be used to make a positive impact on agency, emotion regulation, learning relationships, and effective learning strategies, which are addressed in a wide range of higher education studies in academic performance (Michael and Olive, 2005; Ahmed and Bruinsma, 2006; Villavicencio and Bernardo, 2013). The coaching program could be integrated flexibly into different schools and psychology practices to improve students' performance and attainment. Particularly, introducing mindfulness-based methods and reflective writing would support students to become more self-acceptive, reduce boredom and anxiety, and enhance learning experiences that would increase the chance of academic success (Ruthig et al., 2008).

## Author contributions

QW, HL, and WP contributed to the conception and design of the research. QW, YL, and ZX contributed to empirical work, including data collection and analysis. QW, YL, and ZX drafted the work. QW, HL, YL, ZX, and WP revised the work critically for important intellectual content. All the authors offered final approval of the version to be published, and agreed to be accountable for all aspects of the work in ensuring that questions related to the accuracy or integrity of any part of the work are appropriately investigated and resolved.

### Conflict of interest statement

The authors declare that the research was conducted in the absence of any commercial or financial relationships that could be construed as a potential conflict of interest.
